# Biological correlates associated with high-risk breast cancer patients identified using a computational method

**DOI:** 10.1038/s41523-025-00725-y

**Published:** 2025-01-29

**Authors:** Jung Hun Oh, Fresia Pareja, Rena Elkin, Kaiming Xu, Larry Norton, Joseph O. Deasy

**Affiliations:** 1 Department of Medical Physics, Memorial Sloan Kettering Cancer Center, New York, NY USA; 2 Department of Pathology and Laboratory Medicine, Memorial Sloan Kettering Cancer Center, New York, NY USA; 3 Department of Applied Mathematics and Statistics, Stony Brook University, Stony Brook, NY USA; 4 Department of Medicine, Memorial Sloan Kettering Cancer Center, New York, NY USA

**Keywords:** Cancer genomics, Breast cancer

## Abstract

Using a novel unsupervised method to integrate multi-omic data, we previously identified a breast cancer group with a poor prognosis. In the current study, we characterize the biological features of this subgroup, defined as the high-risk group, using various data sources. Assessment of three published hypoxia signatures showed that the high-risk group exhibited higher hypoxia scores (*p* < 0.0001 in all three signatures), compared to the low-risk group. Our analysis of the immune cell composition using CIBERSORT and leukocyte fraction showed significant differences between the high and low-risk groups across the entire cohort, as well as within PAM50 subtypes. Within the basal subtype, the low-risk group had a statistically significantly higher spatial fraction of tumor-infiltrating lymphocytes (TILs) compared to the high-risk group (*p* = 0.0362). Our findings indicate that this subgroup with poor prognosis is driven by a distinct biological signature with high activation of hypoxia-related genes as well as a low number of TILs.

## Introduction

Breast cancer is a highly heterogeneous disease, and encompasses multiple subtypes based on diverse molecular and histological backgrounds^1–3^. Although widely used for the classification of breast cancer, intrinsic subtypes are not significantly associated with prognosis and response to therapies^4,5^. A number of studies have been conducted to identify novel subgroups associated with poor prognosis outcomes in breast cancer using various genetic and genomic data^6–9^.

We previously developed *aWCluster*, a network-based method that integrates multi-omic data on a graph of cancer-related genes using optimal mass transport (OMT) to identify subgroups^10^. OMT provides a mathematical solution for assessing the similarity or distance between two samples by determining the optimal transportation mapping between mass distributions supported on a feature interaction network^11^. In the aWCluster study, mass distributions were constructed from integrated multi-omic data and the OMT approach was applied on a protein-protein interaction network. Hierarchical clustering was then applied to the resulting Wasserstein distance matrix to identify subgroups. The application of this unsupervised approach to multi-omic data, including gene expression, copy number alteration (CNA), and DNA methylation from The Cancer Genome Atlas Breast Invasive Carcinoma (TCGA-BRCA) dataset, identified a remarkably poor survival breast cancer group. Gene ontology analysis revealed that the subgroup was enriched in hypoxia-related biological pathways. aWCluster works by quantifying large-scale similarities among genes represented in a biological network using integrated multi-omic data. This finding provides a strong rationale to further analyze the resulting subgroup, which may possibly motivate altered clinical care.

Here, we investigate biological correlates of the poor prognosis breast cancer group, by analyzing mutational data, hypoxia signatures, immune cell abundance, leukocyte fraction, and spatial fraction of tumor-infiltrating lymphocytes (TILs) estimated on pathology images.

## Results

### Patients’ characteristics

Table 1 shows the evaluable cohort size in PAM50 subtypes and risk groups for each data type analyzed in the current study. Table 2 shows the clinical characteristics of the patients. Overall survival time was significantly lower in the high-risk group with a median 20.4 months compared to 28.8 months in the low-risk group (Wilcoxon rank-sum *p* = 0.0353). A higher rate of individuals (35.9% and 32.1%) in the high-risk group belonged to the luminal B and basal subtypes compared to those (18.0% and 16.2%) in the low-risk group (Fisher’s exact *p* = 7.7E−7). The ER status did not significantly differ between the high and low-risk groups (*p* = 0.3842). However, the low-risk group had a higher rate of PR-positive breast cancer (68.8%) compared to the high-risk group (53.1%) with *p* = 0.0267 and the high-risk group had a higher rate of HER2-positive breast cancer (28.6%) compared to the low-risk group (12.8%) with *p* = 0.0316. Additionally, the high-risk group was enriched for T2 (79.3%) and stage II tumors (69.2%) compared to the low-risk group (56.0% and 56.3% with *p* = 0.0008 and 0.0011, respectively). Basal subtype was enriched in African American individuals (37.4%), compared to Asian (8.1%) and White (12.9%) individuals with *p* = 5.6E−10. Furthermore, a higher rate of African American individuals belonged to the high-risk group (11.5%) than Asian (8.1%) and White (5.8%) individuals with *p* = 0.0527 (Supplementary Table 1).

**Table 1:** The evaluable cohort size for each data type including CIBERSORT score, somatic mutation, hypoxia signature, estimated spatial fraction of tumor-infiltrating lymphocytes, and leukocyte fraction data

Data type	Group	Luminal A	Luminal B	HER2	Basal	Normal-like	Total
Survival status and CIBERSORT	High risk	10	19	6	17	1	53
	Low risk	376	121	37	109	30	673
Somatic mutation	High risk	8	19	4	16	1	48
	Low risk	335	116	36	98	27	612
Hypoxia	High risk	10	19	6	17	1	53
	Low risk	375	120	37	108	30	670
Estimated spatial fraction of TILs	High risk	10	18	6	14	0	48
	Low risk	312	108	34	90	25	569
Leukocyte fraction	High risk	9	18	6	14	0	47
	Low risk	308	108	34	87	25	562

*TILs* Tumor-infiltrating lymphocytes.

**Table 2:** Clinical characteristics. Statistical analysis was performed using Wilcoxon rank-sum test and Fisher’s exact test for continuous and categorical variables, respectively. Cases with X stage in T, N, and M or NA were excluded in the statistical analysis

Characteristics		High risk group (*N* = 53)	Low risk group (*N* = 673)	*p*-value
Diagnosis age (Median)		59	58	0.9565
Survival time (Median)		20.4	28.8	0.0353
Vital status	Living	43	617	0.0211
	Deceased	10	56	
Race	Asian	3	34	0.0527
	African American	16	123	
	White	31	504	
	Other/NA	3	12	
PAM50 subtype	Luminal A	10	376	7.7E−7
	Luminal B	19	121	
	HER2	6	37	
	Basal	17	109	
	Normal-like	1	30	
ER status	Negative	14	143	0.3842
	Positive	36	491	
	NA	3	39	
PR status	Negative	23	197	0.0267
	Positive	26	435	
	NA	4	41	
HER2 status	Equivocal	9	118	0.0316
	Indeterminate	1	9	
	Negative	20	352	
	Positive	12	70	
	NA	11	124	
T	T1	4	178	0.0008
	T2	42	376	
	T3	4	98	
	T4	3	19	
	TX	0	2	
N	N0	19	302	0.5169
	N1	23	230	
	N2	6	86	
	N3	4	47	
	NX	1	8	
M	cM0	0	3	0.5529
	M0	34	542	
	M1	1	9	
	MX	18	119	
Stage	I	0	111	0.0011
	II	36	374	
	III	15	172	
	IV	1	7	
	Other/NA	1	9	

*ER* estrogen receptor, *PR* progesterone receptor.

### Differential mutation analysis

**Fig. 1:**
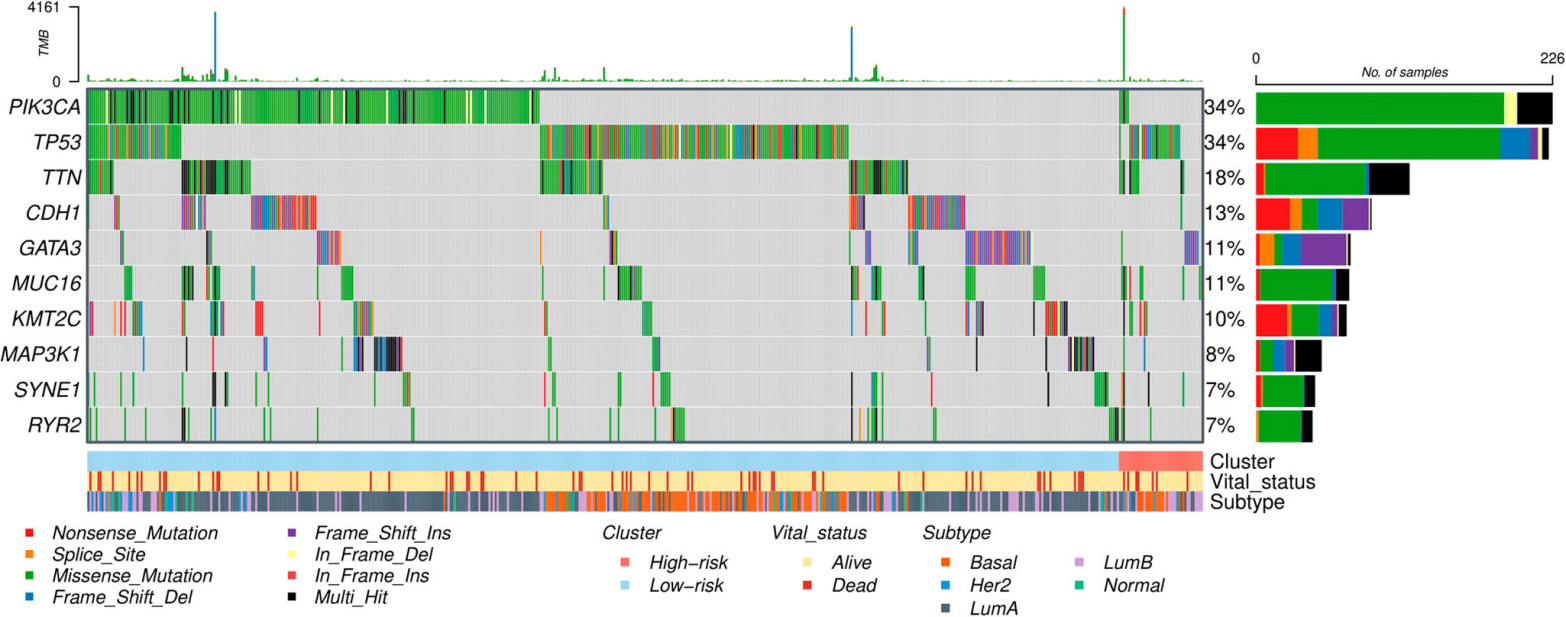
Oncoprint of the top ten most frequently mutated genes in the TCGA-BRCA. The three bars in the bottom indicate the breast cancer risk groups (high [*N* = 48] and low [*N* = 612]), survival status, and PAM50 subtypes, respectively.

Somatic mutation data were available for 660 breast cancer cases from the TCGA, including 48 and 612 cases in the high and low-risk groups, respectively. Differential mutation analysis showed that *PIK3CA* and *TP53* were the top two most frequently mutated genes in the entire cohort (each 34%; Fig. 1). Remarkably, the mutation rates for these two genes were significantly different between the high and low-risk groups; the low-risk group had a higher mutation rate (36.1%, 221/612 vs 10.4%, 5/48; Fisher’s exact *p* = 0.0002) in *PIK3CA*, whereas the high-risk group had a higher mutation rate (54.2%, 26/48 vs 32.2%, 197/612; *p* = 0.0038) in *TP53*. In addition, mutations in both *PIK3CA* and *TP53* were found to be mutually exclusive with *GATA3* as well as *CDH1* mutations (McNemar *p* < 0.0001). The differential mutation analysis was also conducted, comparing mutation rates between the high and low-risk groups within each specific PAM50 subtype. We focused on the basal and luminal B subtypes given their high rates in the high-risk group, representing 33.3% (*N* = 16) and 39.6% (*N* = 19) of cases, respectively. In the luminal B subtype, mutations in *PIK3CA* were statistically significantly more frequent in the low-risk group (31.9%, 37/116) compared to the high-risk group (5.3%, 1/19; *p* = 0.0142; Supplementary Fig. 1). When the basal and luminal B subtypes were combined, the mutational rate difference in *PIK3CA* was marginally significant between the high (5.7%, 2/35) and low (18.7%, 40/214) risk groups (*p* = 0.0848). We also assessed differences in tumor mutational burden (TMB) and mutations of two DNA repair genes (*BRCA1* and *BRCA2*) between the high and low-risk groups but found no significant differences.

### Survival analysis

**Fig. 2:**
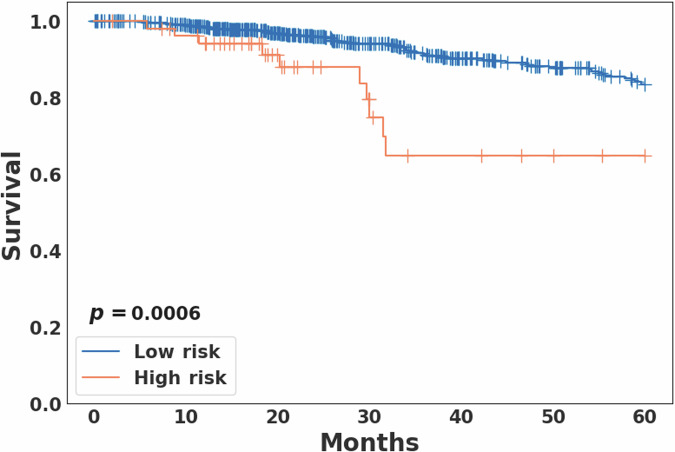
Kaplan-Meier survival analysis between the high (*N* = 53) and low (*N* = 673) risk breast cancer groups. For the four breast cancer subgroups determined from the previous aWCluster analysis^10^, the distinctly poor survival group was denoted as the high risk group and the other three subgroups were merged and denoted as the low risk group. The overall survival rates were significantly different between the two groups (log-rank *p* = 0.0006).

As in the previous study^10^, the follow-up time was truncated at 5 years. In the current study, for the four breast cancer subgroups determined from the previous study, the distinctly poor survival group was denoted as the high-risk group and the other three subgroups were merged and denoted as the low-risk group. Kaplan-Meier survival analysis revealed a statistically significant difference in overall survival between the high (*N* = 53) and low-risk (*N* = 673) groups (log-rank *p* = 0.0006; Fig. 2). The PAM50-based subtype analysis showed marginal statistical significance (*p* = 0.0771) between the high and low-risk groups in the basal subtype, and a non-significant difference (*p* = 0.1720) in the luminal B subtype (Supplementary Fig. 2). However, when the basal and luminal B subtypes were combined, the survival rates between the high and low risk groups were significantly different with *p* = 0.0214 (Supplementary Fig. 2).

### Comparison of hypoxia signature scores

**Fig. 3:**
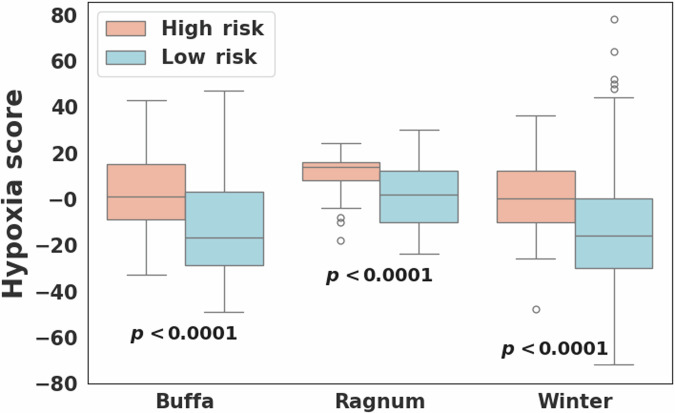
Hypoxia scores for the high and low risk groups. Comparison of hypoxia scores computed for three different hypoxia signatures (Buffa^26^, Ragnum^27^, and Winter^28^) between the high (*N* = 53) and low (*N* = 670) risk breast cancer groups.

**Fig. 4:**
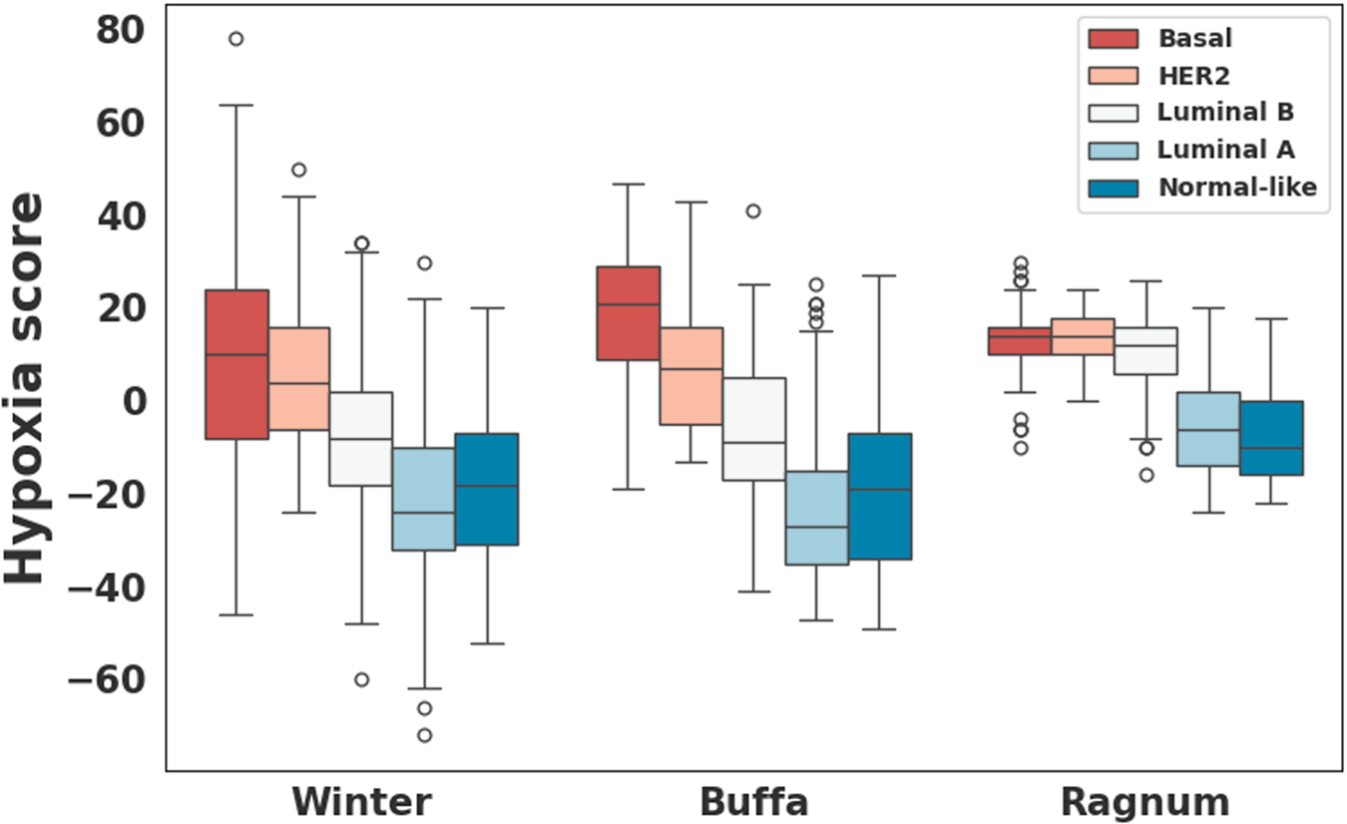
Hypoxia scores for the PAM50-based subtypes. Comparison of hypoxia scores computed for three different hypoxia signatures (Buffa^26^, Ragnum^27^, and Winter^28^) among the PAM50-based subtypes.

Hypoxia scores were available for 723 breast cancer cases including 53 and 670 in the high and low risk groups, respectively. For all three hypoxia signatures, the high-risk group showed statistically significantly higher hypoxia scores compared to the low-risk group (Wilcoxon rank-sum *p* < 0.0001; Fig. 3). In PAM50-based subtype analysis, hypoxia scores differed significantly among the five subtypes, with the basal and HER2 subtypes exhibiting higher scores compared to other subtypes for all three hypoxia signatures (Kruskal–Wallis *p* < 0.0001; Fig. 4). However, no statistically significant difference in hypoxia scores was observed between the high and low-risk groups within each subtype. Notably, breast cancers from African American individuals exhibited significantly higher hypoxia scores (average scores: 0.4, 5.3, and -2.5 in hypoxia signatures from Buffa, Ragnum, and Winter, respectively) compared to other races (Buffa: -13.6, *p* = 1.7E−11; Ragnum: 0.8, *p* = 2.0E−4; Winter: -14.3, *p* = 9.9E−9).

### CIBERSORT analysis to compare immune cell abundance

**Fig. 5:**
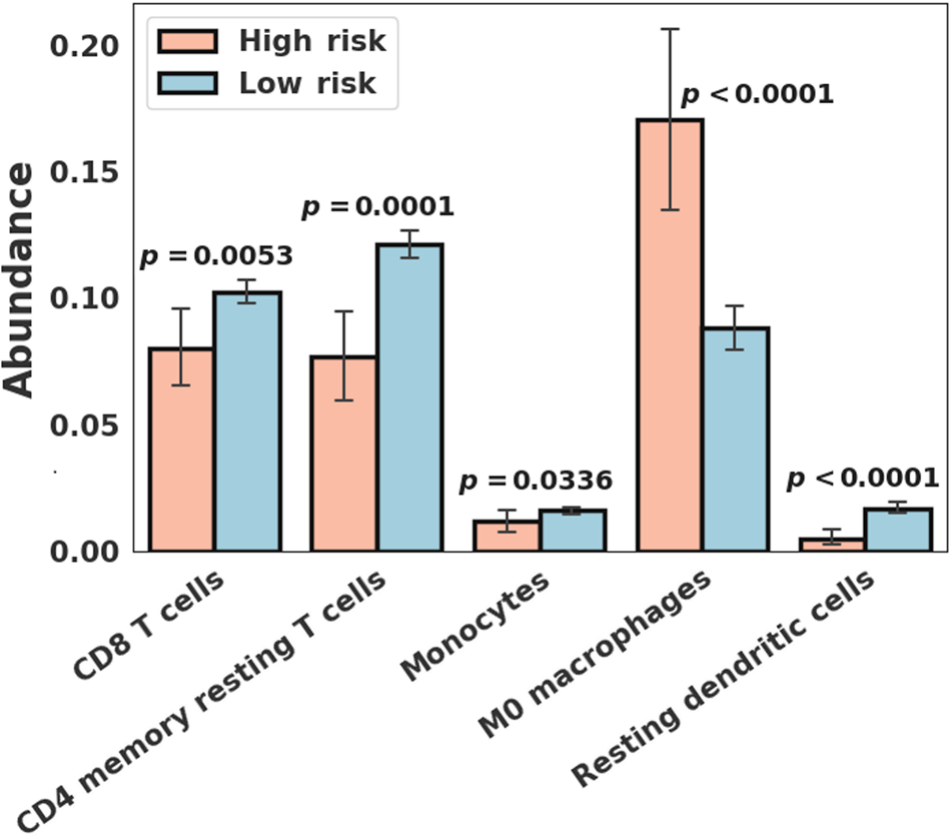
Immune cell type abundance for the high and low risk groups. Immune cell type abundance showing statistically significant differences (*p* < 0.05) between the high (*N* = 53) and low (*N* = 673) risk breast cancer groups.

CIBERSORT scores were available for all 726 breast cancer cases. CIBERSORT analysis showed a statistically significantly higher abundance of CD8 T cells (Wilcoxon rank-sum *p* = 0.0053), CD4 memory resting T cells (*p* = 0.0001), monocytes (*p* = 0.0336), and resting dendritic cells (*p* < 0.0001) in the low risk group compared to the high risk group (Fig. 5). M0 macrophages showed a statistically significant greater abundance in the high risk group compared to the low risk group (*p* < 0.0001). In PAM50-based subtype analysis, the basal subtype showed a statistically significantly higher abundance of CD4 memory resting T cells (*p* = 0.0021) and regulatory Tregs T cells (*p* = 0.0030) in the low risk group and M0 macrophages (*p* = 0.0491) in the high risk group. The luminal B subtype showed a statistically significant greater abundance of resting dendritic cells (*p* = 0.0048) in the low risk group. When the basal and luminal B subtypes were combined, four immune cell types, including CD4 memory resting T cells (*p* = 0.0008), regulatory Tregs T cells (*p* = 0.0098), M1 macrophages (*p* = 0.0104), and resting dendritic cells (*p* = 0.0011), showed a statistically significantly higher abundance in the low risk group and M0 macrophages (*p* = 0.0094) in the high risk group (Table 3 and Supplementary Fig. 3).

**Table 3:** Immune cell types (with *p*-values) with statistically significant differences (*p* < 0.05) between the high (*N* = 53) and low (*N* = 673) risk breast cancer groups across the entire cohort and within each PAM50 subtype

Immune cell type	Entire cohort (*N* = 726)	Basal (*N* = 126)	Luminal B (*N* = 140)	Basal + Luminal B (*N* = 266)
CD8 T cells	0.0053			
CD4 memory resting T cells	0.0001	0.0021		0.0008
Regulatory Tregs T cells		0.0030		0.0098
M0 macrophages	<0.0001	0.0491		0.0094
M1 macrophages				0.0104
Resting dendritic cells	<0.0001		0.0048	0.0011
Monocytes	0.0336			

### Analysis of leukocyte fraction and tumor-infiltrating lymphocytes

Leukocyte fraction data were available for 609 breast cancer cases, including 47 and 562 in the high and low-risk groups, respectively. The leukocyte fraction had statistical significance on the entire cohort (Wilcoxon rank-sum *p* = 1.3E−5) as well as PAM50 subtypes including the basal (*p* = 4.2E−7), luminal B (*p* = 0.0214), and combined basal and luminal B subtypes (*p* = 6.0E−8) with higher leukocyte fractions in the low-risk group.

**Fig. 6:**
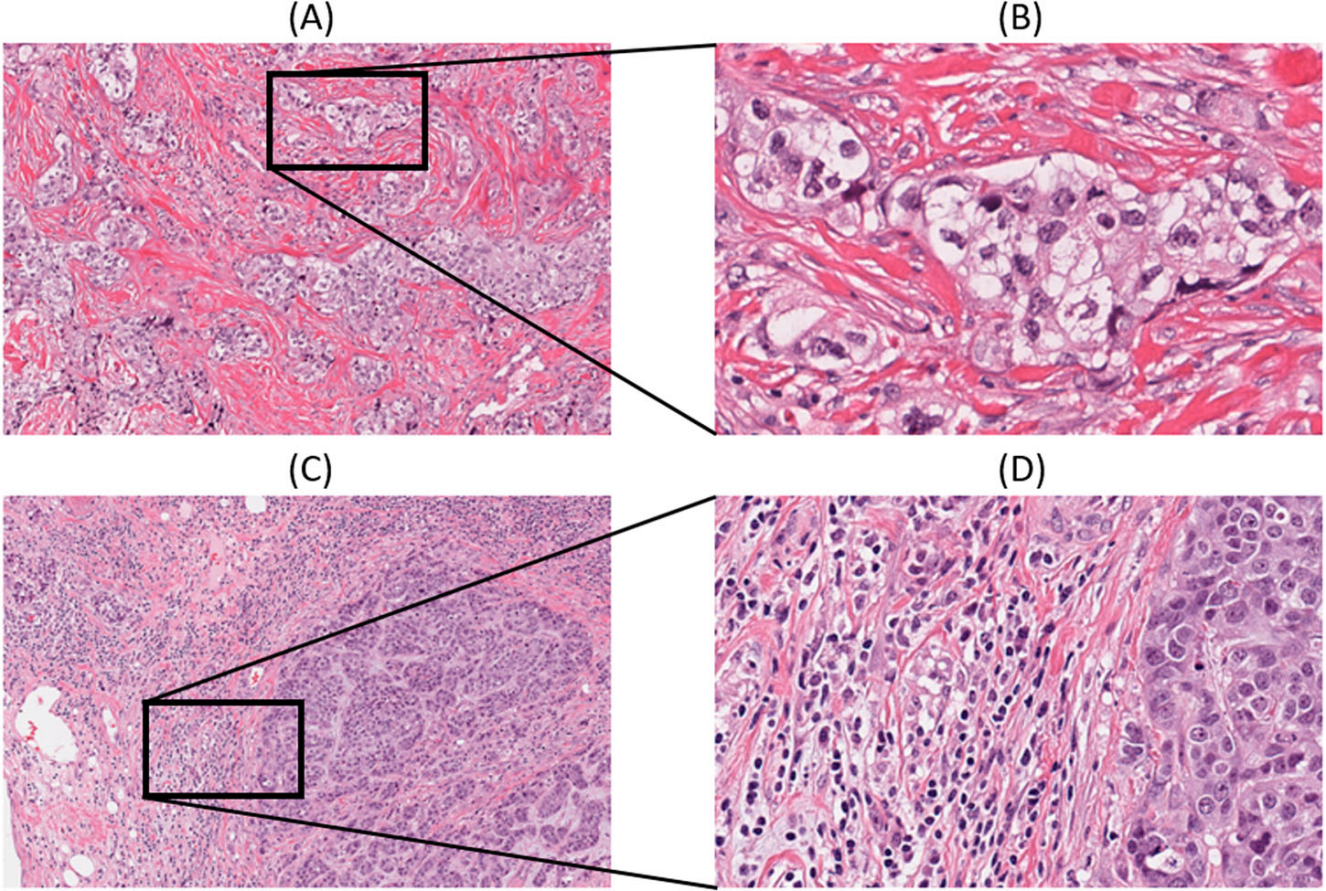
Representative micrographs of basal breast cancer cases included in the current study. Images were obtained from https://cancer.digitalslidearchive.org/; **A** low-power and **B** high-power micrographs of the high risk group case (TCGA-A7-A26I) with an estimated spatial fraction of TILs of 0.1%, depicting rare TILs and (**C**) low power and (**D**) high power micrographs of the low-risk group case (TCGA-E2-A1B6) with an estimated spatial fraction of TILs of 20.0%, which depicts a high extent of TILs. The spatial fraction of TILs was obtained from the study conducted by Saltz et al.^12^.

The spatial fraction of TILs estimated on pathology images was available for 617 breast cancer cases including 48 and 569 in the high and low risk groups, respectively^12^. The spatial fraction of TILs was not significantly different between the high and low-risk groups in the entire cohort. In PAM50-based subtype analysis, a statistically significantly higher spatial fraction of TILs was found in the low-risk group compared to the high-risk group within the basal subtype (Wilcoxon rank-sum *p* = 0.0361). Figure 6 illustrates two representative micrographs with the basal subtype included in the current study. Figure 6A displays the case (TCGA-A7-A26I) in the high-risk group with an estimated spatial fraction of TILs of 0.1% and rare stromal TILs (<1%) by histopathologic examination whereas Fig. 6B displays the case (TCGA-E2-A1B6) in the low-risk group with an estimated spatial fraction of TILs of 20.0% and a high extent of stromal TILs (60%).

In the high risk group, CNA and DNA methylation data were correlated with the leukocyte fraction and spatial fraction of TILs. A number of genes on DNA methylation data were found to be highly correlated with the leukocyte fraction. Pathway analysis was conducted on the top ranked 100 genes, revealing allograft rejection as a significantly enriched pathway (false discovery rate [FDR] = 2.5E-7).

## Discussion

In our previous study, we developed a network-based multi-omic data integration method called aWCluster applied to RNA-Seq, CNA, and DNA methylation data^10^. By applying this unsupervised approach to the TCGA-BRCA, we identified a high-risk group with distinct biology and found that hypoxia-related pathways are associated with poor prognosis, using downstream bioinformatics approaches on RNA-Seq data. In the current study, we further investigated the biological features relevant to the high-risk group identified in the previous work, by analyzing various biological data including somatic mutation, hypoxia signatures, immune cell abundance, leukocyte fraction, and spatial fraction of TILs estimated on pathology images.

*PIK3CA* and *TP53* were found to be the two most frequently mutated genes in the TCGA-BRCA, as well as differentially mutated genes between the high and low-risk groups. *PIK3CA* is the most commonly mutated gene in breast cancer, occurring in up to 40% of ER+/HER2- breast tumors^13^. Mutations in *PIK3CA* may lead to the activation of proliferation and apoptosis^14^. *TP53* mutations are also common in breast cancer with a much higher mutation rate in the basal-like subtype and its mutation is known to be associated with more aggressive tumor characteristics^15–18^. In our analysis, the low-risk group had a higher mutation rate (36.1% vs 10.4%; Fisher’s exact *p* = 0.0002) in *PIK3CA* whereas the high-risk group had a higher mutation rate (54.2% vs 32.2%; *p* = 0.0038) in *TP53*. In particular, for the luminal B subtype, *PIK3CA* showed a statistically significant difference between the high and low-risk groups with *p* = 0.0142. Mutations were observed to be unevenly distributed across PAM50 subtypes. For instance, *PIK3CA* (3.2%), *CDH1* (0.8%), and *GATA3* (0%) were less frequently mutated in the basal subtype. In PAM50-based subtype analysis, we more focused on the basal and luminal B subtypes because of their high rates in the high-risk group (in total, 72.9%). It is worth noting that previous studies have reported that basal-like and triple-negative tumors are associated with aggressive clinical behavior and poor outcomes^19^ and luminal B tumors have a relatively higher grade and lead to worse prognosis compared to luminal A tumors^20,21^.

Individuals in the high-risk group were enriched for the basal and luminal B subtypes with a higher rate of HER2-positive and a lower rate of PR-positive breast cancer compared to the low-risk group. Not surprisingly, the high-risk group exhibited a lower proportion of T1 and stage I tumors (7.6% and 0%) relative to the low-risk group (26.5% and 16.7%).

Kaplan-Meier analysis showed a statistically significant survival difference between the high and low-risk groups across the entire cohort, as well as when combining the basal and luminal B subtypes. Three published hypoxia signatures were assessed, resulting in all statistically significant differences with the high-risk group having higher scores, but no statistically significant difference was observed within each PAM50 subtype. Notably, hypoxia scores differed significantly among the PAM50 subtypes, with the basal and HER2 subtypes exhibiting higher scores compared to other subtypes. A recent study reported that the VEGF-hypoxia signature is highly enriched in the basal subtype compared to other subtypes, with higher expression in women of African ancestry compared to While women, and is likely to be associated with poor outcomes^22^. This is in line with our findings that showed that African American individuals had a higher rate of basal subtype breast cancer with significantly higher hypoxia scores compared to other races. Significant differences in various immune cell types and leukocyte fraction were observed between the high and low-risk groups on the entire cohort, as well as within the different PAM50 subtypes, suggesting differences in the tumor immune microenvironment. In addition, within the basal subtype we observed a statistically significant difference in the spatial fraction of TILs estimated on pathology images between the high and low-risk groups, with a higher spatial fraction of TILs in the low-risk group.

These findings indicate that breast cancers with poor prognosis are associated with differential mutation rates in key genes, as well as higher hypoxia levels and differences in the tumor immune microenvironment such as lower TIL infiltration. We plan to further investigate biological correlates associated with poor outcomes in the unfavorable breast cancer subtype with the ultimate aim of improving clinical decision-making including therapeutic choices.

## Methods

### Subgroups in breast cancer

**Fig. 7:**
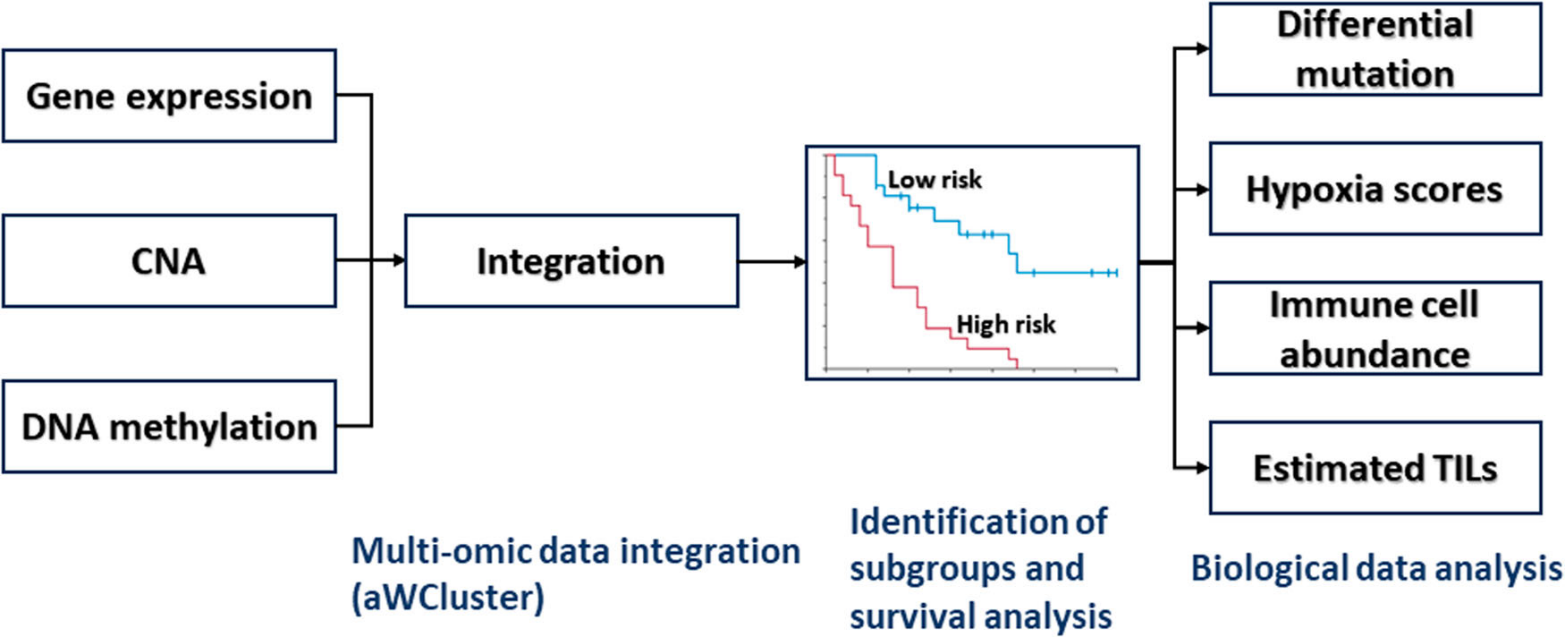
Data analysis pipeline and biological data. CNA copy number alteration, TILs tumor-infiltrating lymphocytes.

In the current study, we analyzed the TCGA-BRCA (*N* = 726). Here, we denoted the distinctly poor survival breast cancer group identified in our previous study^10^ as the “high risk group” (*N* = 53, 7.3%); all others were denoted as the “low risk group” (*N* = 673, 92.7%). PAM50 subtype information was obtained from two published breast cancer studies^23,24^. Figure 7 illustrates the data analysis pipeline and biological data analyzed using bioinformatic and statistical analysis methods. Ethical review was not required because only publicly available data were analyzed.

### Differential mutation

For differential mutation analysis, TCGA pan-cancer mutational profiles with somatic mutation information aggregated at the gene level were downloaded from the Genomic Data Commons (GDC) database (https://gdc.cancer.gov/about-data/publications/mc3-2017)^25^.

### Hypoxia scores

Gene expression-based tumor-hypoxia signatures developed by Buffa et al.^26^, Ragnum et al.^27^, and Winter et al.^28^ were assessed. Hypoxia scores computed on RNA-Seq data from the TCGA-BRCA for the three published signatures were downloaded from the cBioPortal database (https://www.cbioportal.org)^29^.

### Immune cell abundance

TCGA-BRCA CIBERSORT scores that quantify 22 immune cell types using a support vector regression-based deconvolution method were downloaded from the GDC database (https://gdc.cancer.gov/about-data/publications/panimmune)^30^.

### Spatial fraction of tumor-infiltrating lymphocytes

To develop digital-pathology-based diagnostic and prognostic biomarkers, Saltz et al. introduced a deep learning “computational stain” approach on pathology images to estimate the spatial fraction of TILs^12^. Prior to the analysis, each whole-slide image was partitioned into patches. The spatial fraction of TILs was determined by dividing the number of TIL-positive patches by the total number of identified patches on the tissue sample. The estimated spatial fraction of TILs and leukocyte fraction data quantified using DNA methylation arrays were downloaded from the study by Saltz et al.^12^. The spatial fraction of TILs was denoted as the fraction of TIL-positive patches out of the total number of patches within the tissue.

### Statistical analysis

For statistical analysis, differences in these biological data between the high and low risk groups were assessed using Wilcoxon rank-sum test and Fisher’s exact test for continuous and categorical variables, respectively. To investigate mutual exclusivity in mutations between gene pairs of interest, the McNemar test was employed. For survival analysis, Kaplan-Meier analysis with log-rank test was used.

## Supplementary information

Supplementary information

## Data Availability

TCGA pan-cancer mutational profiles were downloaded from the Genomic Data Commons (GDC) database (https://gdc.cancer.gov/about-data/publications/mc3-2017). Hypoxia scores for the three published signatures were downloaded from the cBioPortal database (https://www.cbioportal.org). CIBERSORT scores were downloaded from the GDC database (https://gdc.cancer.gov/about-data/publications/panimmune). The leukocyte fraction and estimated spatial fraction of tumor-infiltrating lymphocytes are available at Supplemental Information of 10.1016/j.celrep.2018.03.086.
