# Supplementary material for: Biological correlates associated with high-risk breast cancer patients identified using a computational method
Source: NPJ Breast Cancer. 2025 Jan 29;11:8. doi: 10.1038/s41523-025-00725-y (PMC11775240; doi:10.1038/s41523-025-00725-y)
Supplement: Supplementary file 1 — Supplementary information [file 41523_2025_725_MOESM1_ESM.pdf]

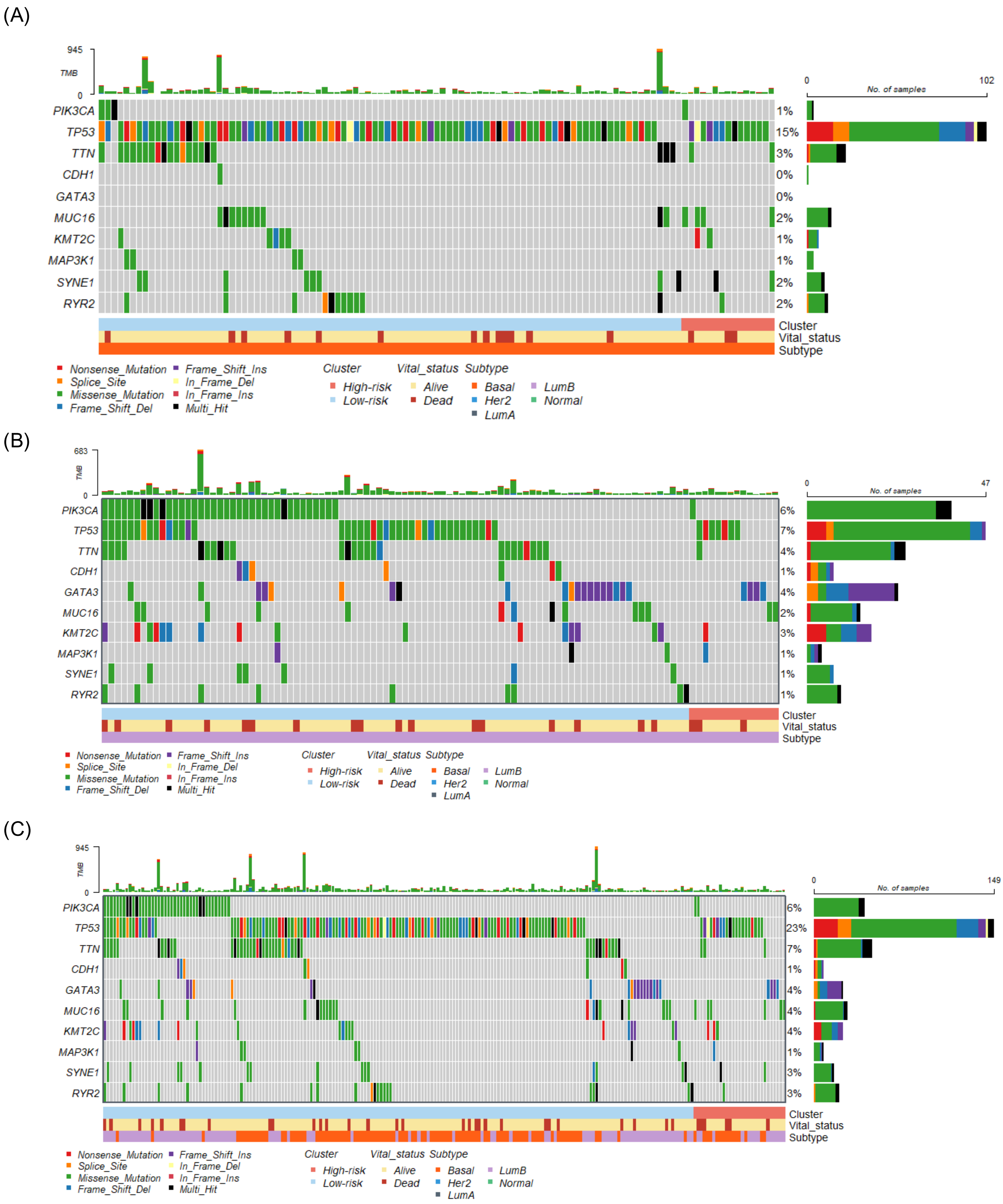

Supplementary Figure 1. Oncoprint of the top ten most frequently mutated genes for (A) basal, (B) luminal B, and (C) basal+luminal B subtypes in the TCGA-BRCA.

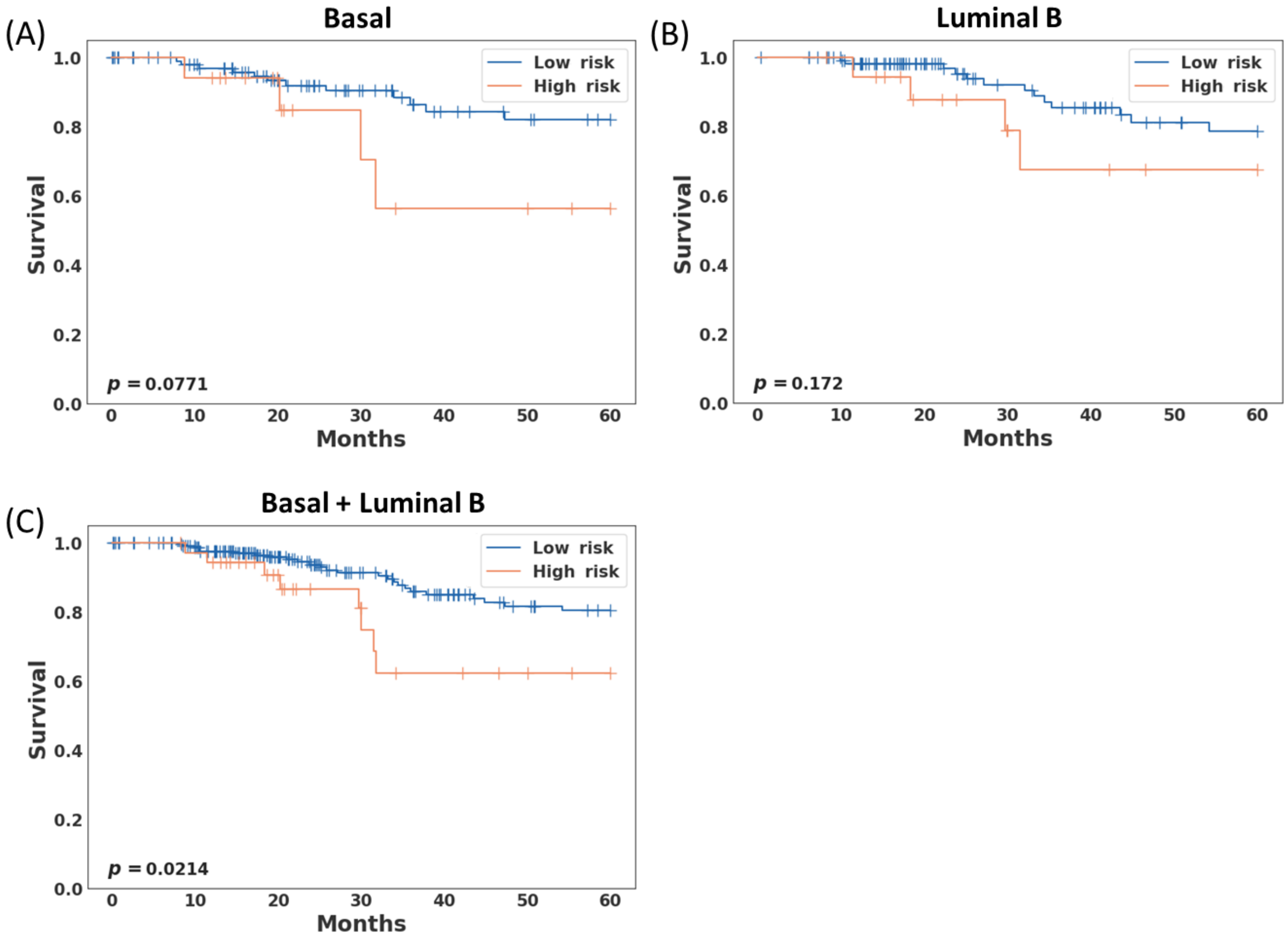

Supplementary Figure 2. Kaplan-Meier survival analysis between the high and low risk breast cancer groups for (A) basal, (B) luminal B, and (C) basal+luminal B subtypes.

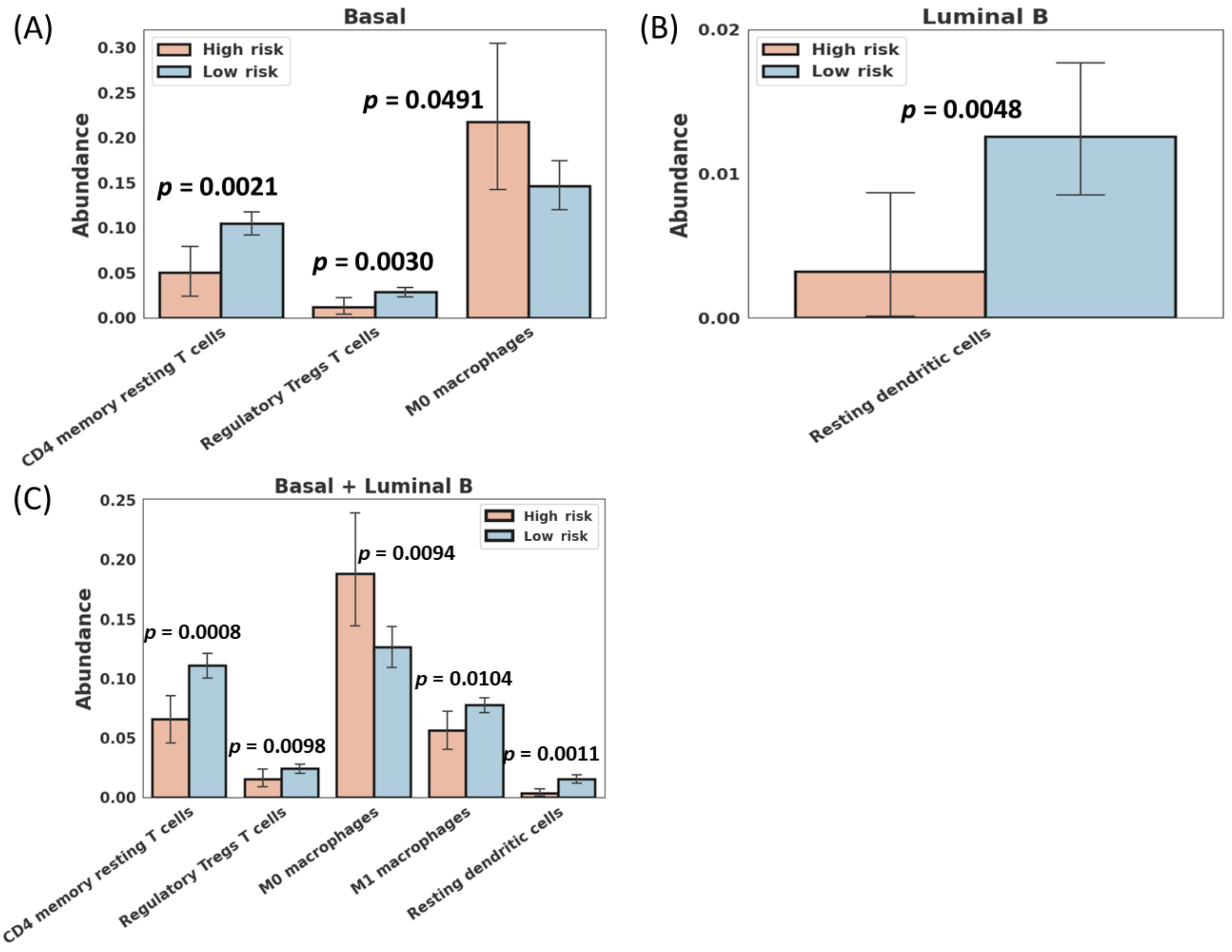

Supplementary Figure 3. Immune cell type abundance showing statistically significant differences ($p < 0.05$) between the high and low risk breast cancer groups for (A) basal, (B) luminal B, and (C) basal+luminal B subtypes.

Supplementary Table 1. The number of cases for each race and PAM50 subtype.

Race	Luminal A	Luminal B	HER2	Basal	Normal-like	Group
Asian	14 (37.84%)	11 (29.73%)	8 (21.62%)	3 (8.11%)	1 (2.70%)	High risk (3, 8.11%) vs Low risk (34, 91.89%)
African American	45 (32.37%)	23 (16.55%)	12 (8.63%)	52 (37.41%)	7 (5.04%)	High risk (16, 11.51%) vs Low risk (123, 88.49%)
White	319 (59.63%)	103 (19.25%)	21 (3.92%)	69 (12.90%)	23 (4.30%)	High risk (31, 5.79%) vs Low risk (504, 94.21%)
Other/NA	8 (53.34%)	3 (20.00%)	2 (13.33%)	2 (13.33%)	0 (0.00%)	High risk (3, 20.00%) vs Low risk (12, 80.00%)
